# Supplementary material for: tstrait: a quantitative trait simulator for ancestral recombination graphs
Source: Bioinformatics. 2024 May 25;40(6):btae334. doi: 10.1093/bioinformatics/btae334 (PMC11784591; doi:10.1093/bioinformatics/btae334)
Supplement: btae334_Supplementary_Data [file btae334_supplementary_data.pdf]

Supplementary Material for tstrait: a quantitative trait simulator for ancestral recombination graphs

1 Supplementary Notes

We present an overview of statistical tests that are conducted during the development process of the `tstrait` package. Three types of tests are conducted to validate the statistical properties of `tstrait`: exact tests, comparison tests, and statistical tests.

1.1 Exact Tests

We simulate effect sizes and phenotypes without environmental noise by using two different quantitative trait simulators, `AlphaSimR` [Gaynor et al., 2021] and `simplePHENOTYPES` [Fernandes and Lipka, 2020], and the simulation framework described in [Zhang et al., 2023]. The simulated effect sizes are directly used in `tstrait` to simulate phenotypes. We show that when traits are influenced with the same causal sites and effect sizes, the simulated genetic values from `tstrait` are identical to the other simulation frameworks.

1.2 Comparison Tests

We simulate phenotypes with environmental noise in `AlphaSimR` [Gaynor et al., 2021], `simplePHENOTYPES` [Fernandes and Lipka, 2020] and the simulation framework described in [Zhang et al., 2023] by using the same parameters that are used in the `tstrait` simulation. By using QQ-plots, we show that the simulated traits from `tstrait` are having similar distributions as the traits simulated from other programs.

1.3 Statistical Tests

Environmental noise and effect sizes are simulated by using various parameters in `tstrait` and their statistical properties are validated by using QQ-pplots.

1.4 Availability of Materials

Code to run the statistical tests is included in the `tstrait` GitHub repository, `https://github.com/tskit-dev/tstrait/blob/main/verification.py`. These tests are used as part of the development process of the `tstrait` package.

2 Supplementary Figures

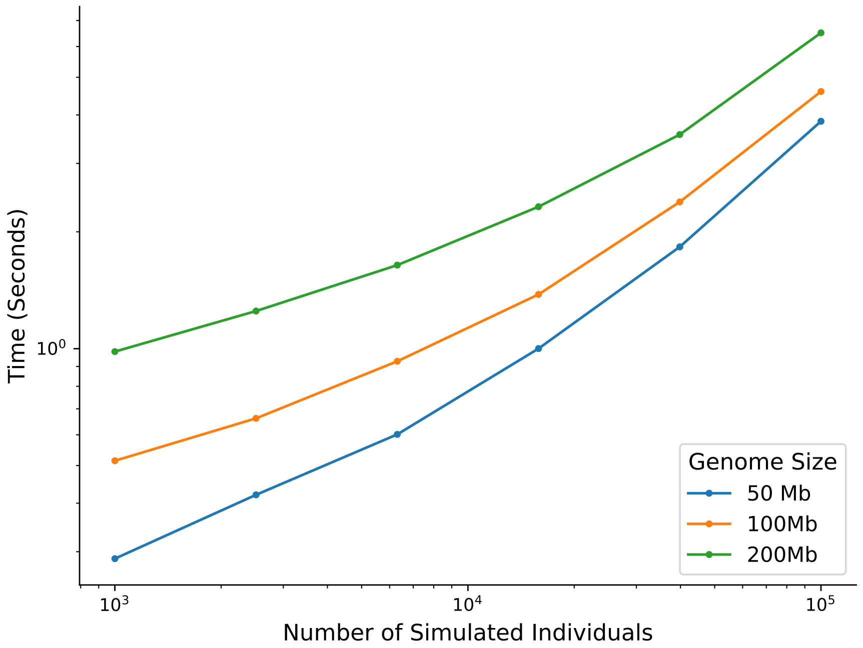

Figure S1: Time taken to simulate quantitative traits with increasing sample size. For each sample size, we simulated an ARG under human-like parameters using the default HomSap demographic model in `stdpopsim`. Each point represents the mean time for 10 independent runs of `tstrait` for a particular ARG. The times reported are the total CPU time required to simulate a quantitative trait with 1000 causal sites, on an Intel(R) Core(TM) i9-11900H CPU and 16 GB of RAM. The trait model is a normal distribution with $\mu = 0$, $\sigma^2 = 1$, $h^2 = 0.3$, and $\alpha = 0$.

References

S. B. Fernandes and A. E. Lipka. simplePHENOTYPES: SIMulation of pleiotropic, linked and epistatic phenotypes. *BMC Bioinform.*, 21(1):1–10, 2020.

R. C. Gaynor, G. Gorjanc, and J. M. Hickey. AlphaSimR: an R package for breeding program simulations. *G3*, 11(2):jkaa017, 2021.

B. C. Zhang, A. Biddanda, Á. F. Gunnarsson, F. Cooper, and P. F. Palamara. Biobank-scale inference of ancestral recombination graphs enables genealogical analysis of complex traits. *Nat. Genet.*, pages 1–9, 2023.
